# Supplementary material for: Inhibition of HDAC6 alters fumarate hydratase activity and mitochondrial structure
Source: Nat Commun. 2025 Jul 28;16:6923. doi: 10.1038/s41467-025-61897-6 (PMC12304134; doi:10.1038/s41467-025-61897-6)

## INHIBITION OF HDAC6 ALTERS FUMARATE HYDRATASE ACTIVITY AND MITOCHONDRIAL STRUCTURE

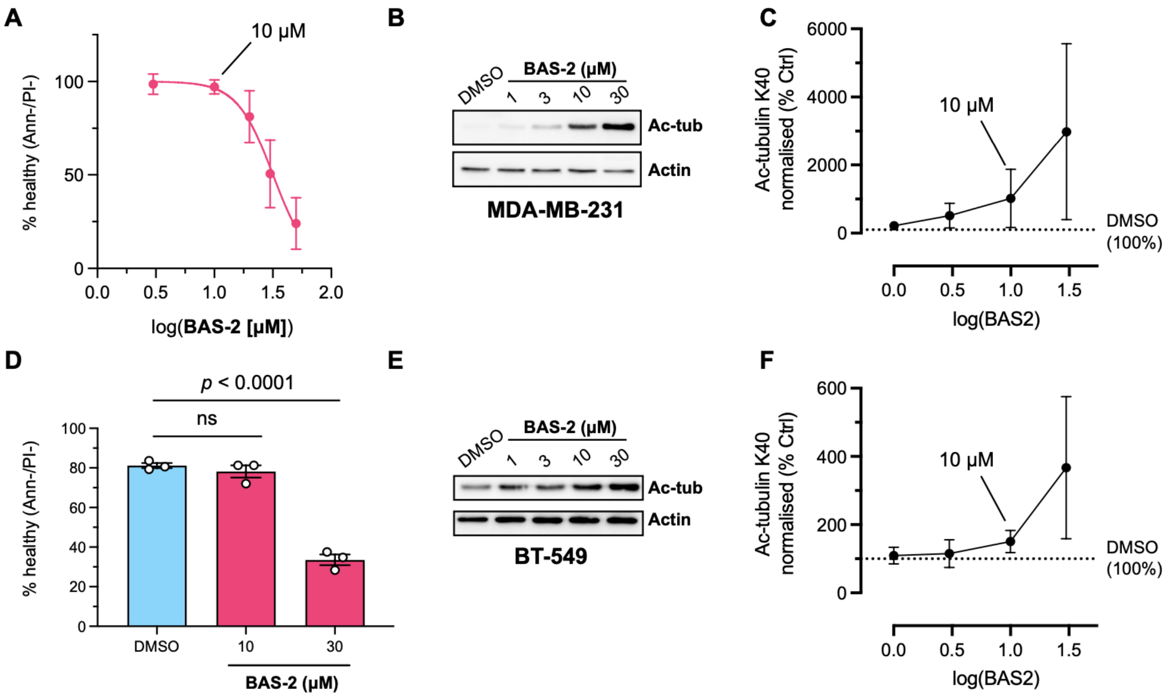


**Supplementary Figure 1. BAS-2 inhibits HDAC6 at sub-lethal doses and results in cell death at higher doses in TNBC cells.** (A) Cell death of MDA-MB-231 cells as measured by Annexin V-FITC and propidium iodide (PI) staining, where double-negative events were designated 'healthy'. Values normalised to DMSO control as 100 % and a dose-response 'inhibitor' variable slope was fitted using Prism (*n* = 3). (B) Representative Western blot of MDA-MB-231 cell lysates after treatment with BAS-2 for 24 h and probed for acetyl-tubulin (K40) as a marker of HDAC6 inhibition. (C) Densitometry for acetylated tubulin normalised to ß-actin from MDA-MB-231 cells (*n* = 3). (D) Cell death in BT-549 cells after 24 h treatment with BAS-2 at 10 or 30 μM as measured by Annexin V-FITC/PI staining. (E) Representative Western blot of BT-549 cells after BAS-2 treatment at the indicated doses (24 h) with acetylated tubulin levels. (F) Densitometry of BT-549 Western images normalised to ß-actin and DMSO control (*n* = 3). DMSO levels are indicated by the dotted line (C and F).

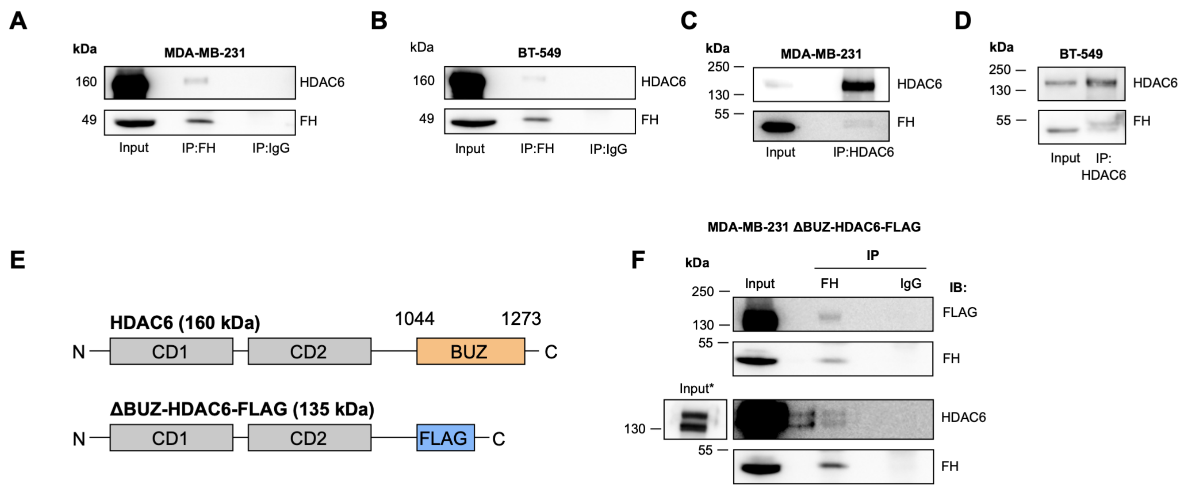


**Supplementary Figure 2. HDAC6 binds FH independent of its ubiquitin-binding domain.** (A) Immunoprecipitation of FH (IP:FH) or IgG control (IP:IgG) in whole-cell lysates of MDA-MB-231 and (B) BT-549 cells and immunoblot (IB) showing HDAC6 binding. (C) Immunoprecipitation of HDAC6 in MDA-MB-231 cells showing FH binding. (D) Immunoprecipitation of HDAC6 in BT-549 cells showing FH binding. (E) Schematic of full-length HDAC6 and FLAG-tagged vector missing the ubiquitin-binding domain, ΔBUZ-HDAC6-FLAG. (F) Immunoprecipitation (IP) of MDA-MB-231 cells stably expressing ΔBUZ-HDAC6-FLAG using either FH of IgG control and immunoblot (IB) for either FLAG (top two panels) or HDAC6 antibody (bottom two panels). Input* shows a lower exposure time.

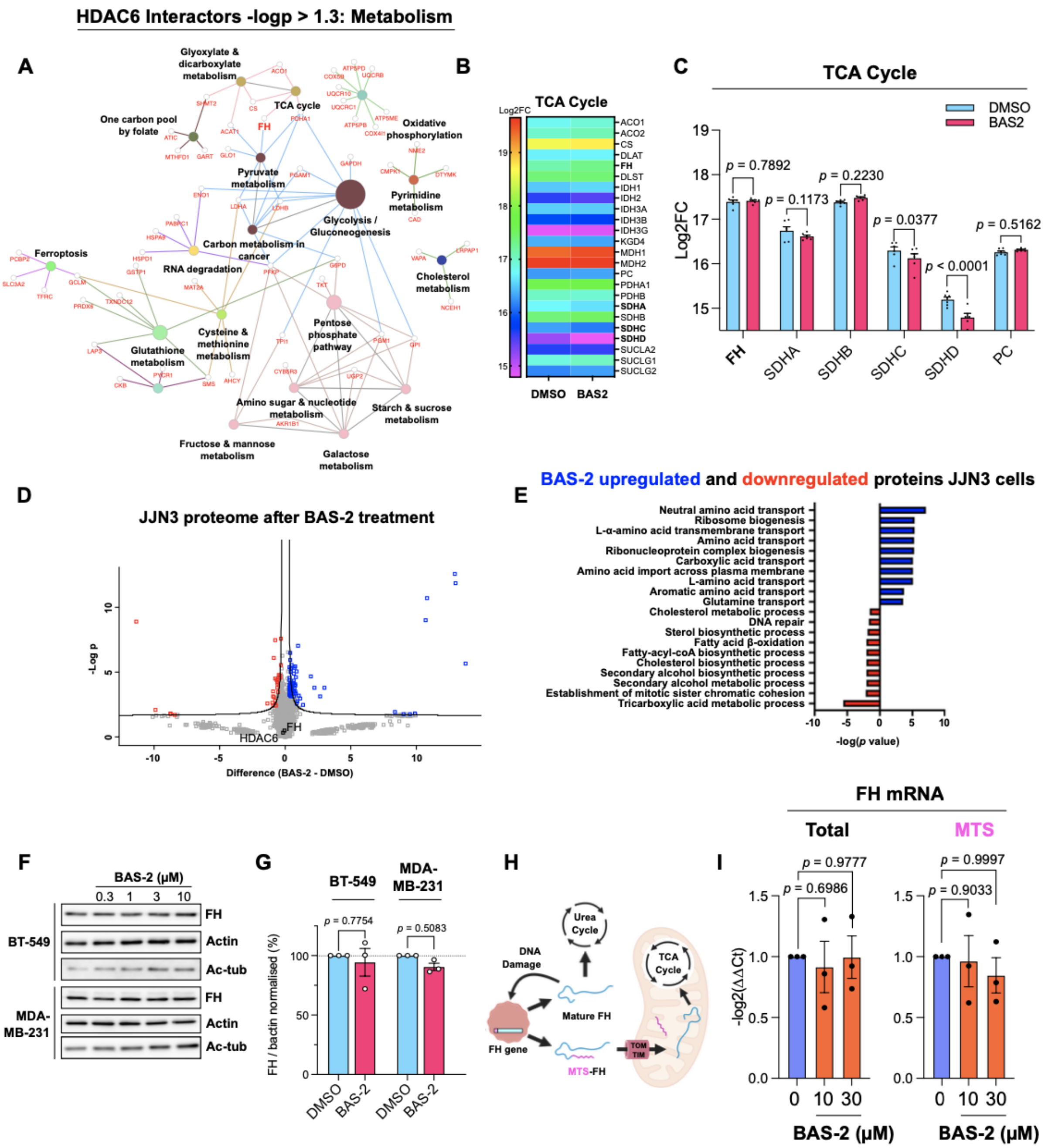


**Supplementary Figure 3. HDAC6 interacts with multiple metabolic proteins and treatment with BAS-2 alters metabolic protein expression of MDA-MB-231 and JJN3 cells** (A) STRING-based visualisation of metabolic pathway KEGG terms and associated HDAC6-interacting proteins above the probability threshold, using HDAC6 immunoprecipitation compared to IgG control. (B) Heat-map showing the relative expression of all TCA cycle enzyme expression after BAS-2 treatment (10 µM) for 24 h in MDA-MB-231 cells. (C) Selected TCA cycle enzyme expression after BAS-2. Points represent technical replicates and significance by multiple t-tests. (D) Volcano plot of upregulated (blue) and downregulated processes in JJN3 cells after BAS-2 treatment. (E) Proteins that were significantly upregulated and downregulated are

graphed by biological processes. (F) Representative Western blots of FH expression and acetyl-tubulin in MDA-MB-231 and BT-549 cells after treatment with BAS-2 for 24 h. (G) Densitometry of FH expression normalised to β-actin. (H) Schematic of the processing of cytosolic FH and FH containing a mitochondrial-targeting signal (MTS). Created in BioRender. Ní Chonghaile, T. (2025) https://BioRender.com/w7lfu9o. (I) mRNA levels of total-FH and mitochondrial-targeting signal-FH (MTS-FH) in MDA-MB-231 cells after 24 h treatment with BAS-2. Significance assessed by t-test.

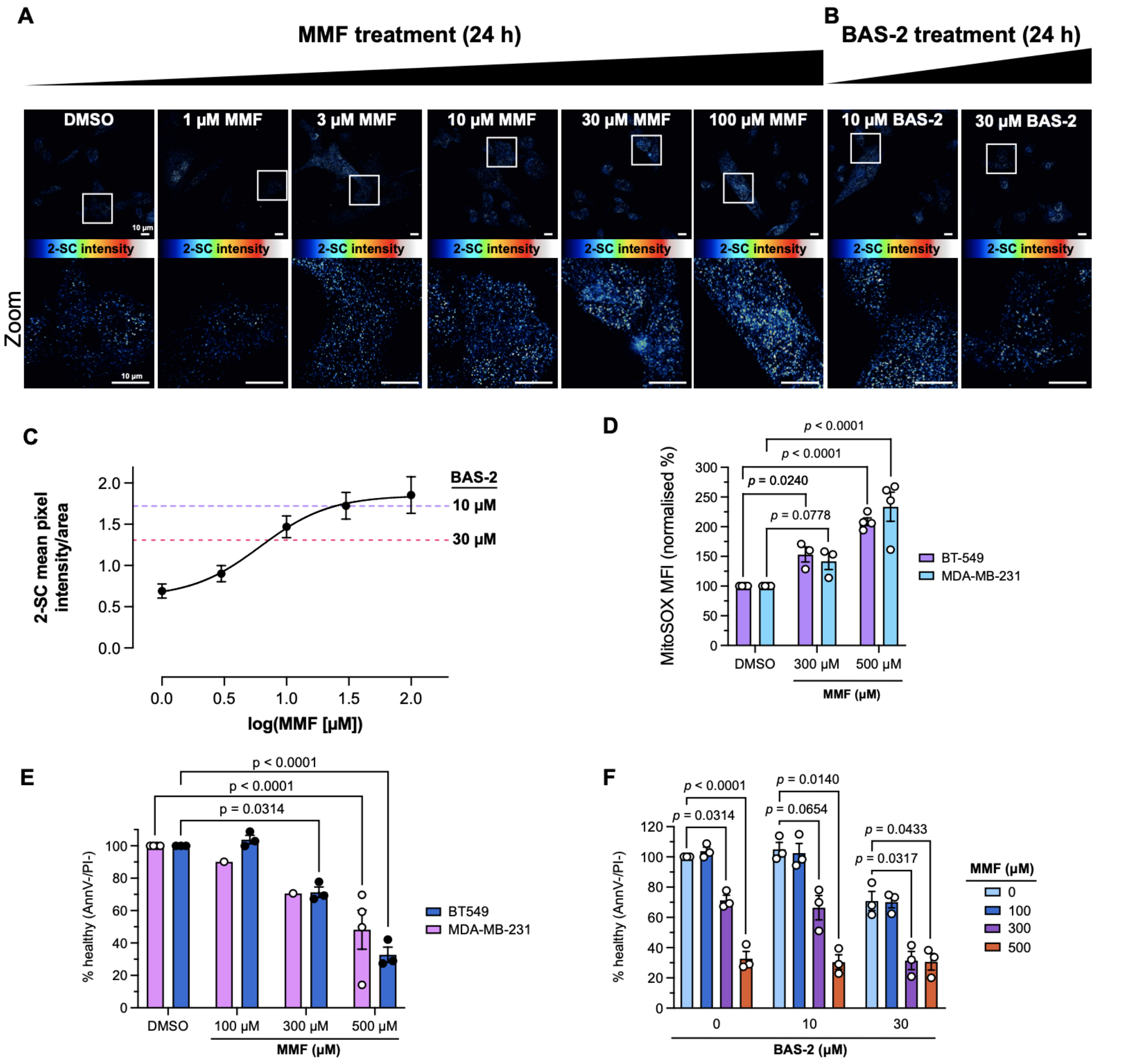


**Supplementary Figure 4. BAS-2 results in increased protein succination.** (A-B) Representative immunofluorescence images of MDA-MB-231 cells after 24 h treatment with BAS-2 or monomethyl fumarate (MMF) using 2-succinocysteine (2-SC) antibody and anti-rabbit Star635P (S635P) secondary to indicate protein succination. Scale bars show 10 µm. Uniformly adjusted 2-SC-S635P intensity is coloured using the 'Royal' colour map in ImageJ/FIJI of the scale (left to right) 0-100 %. (C) Cells were segmented, and mean 2-SC pixel intensity quantified as a proportion of the area quantified. These values were then used to construct the standard curve in (E). Points here represent mean ± SEM from 10-15 cells from one independent experiment. The means of BAS-2 treatment are shown as dotted lines to indicate the equivalent MMF concentration (D) mtROS as indicated by MitoSOX Red staining in response to MMF treatment for 48 h at the doses indicated in either BT-549 or MDA-MB-

231 cells. Values represent mean fluorescence intensity (MFI) ± SEM normalised to DMSO control (*n* = 3-4). (E) Cell death induced by 48 h MMF treatment of BT-549 and MDA-MB-231 cells at the doses indicated, as assessed by Annexin V-FITC/propidium iodide (PI) staining flow cytometry. Values show mean ± SEM normalised to control (*n* = 3 BT-549, *n* = 1-4 MDA-MB-231). (F) Cell death after co-treatment of BAS-2 (24 h) and MMF (48 h) in MDA-MB-231 cells as assessed by Annexin V-FITC/PI flow cytometry.

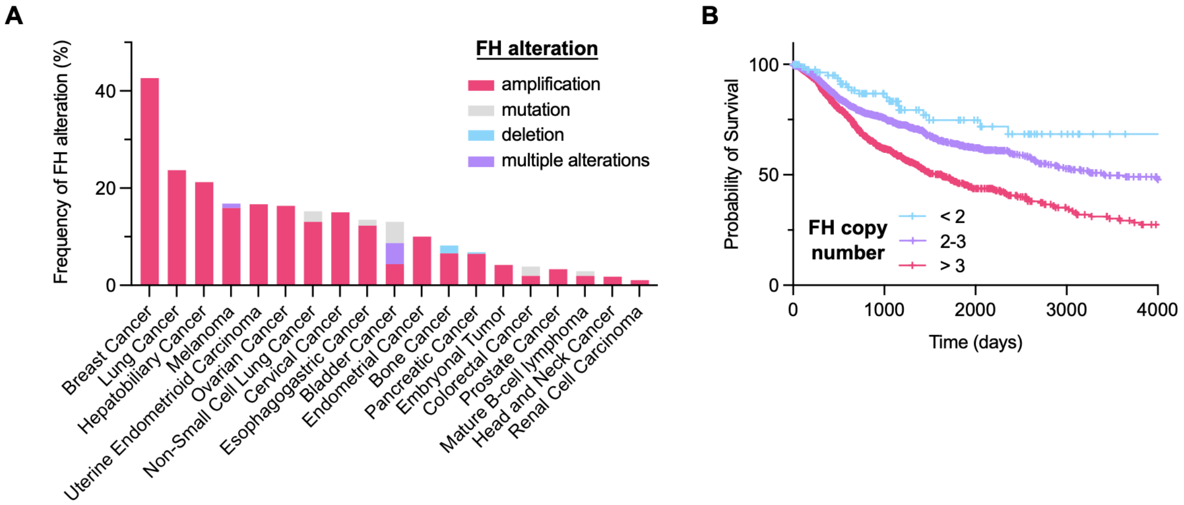


**Supplementary Figure 5. FH amplification is increased in breast cancer and FH copy number correlates with survival.** (A) Frequency of FH gene alterations from the pan-cancer analysis of whole genomes dataset[50] and ordered by cancer subtype. (B) Kaplan-Meier survival curve of cancer patients grouped by level of FH copy number amplification.

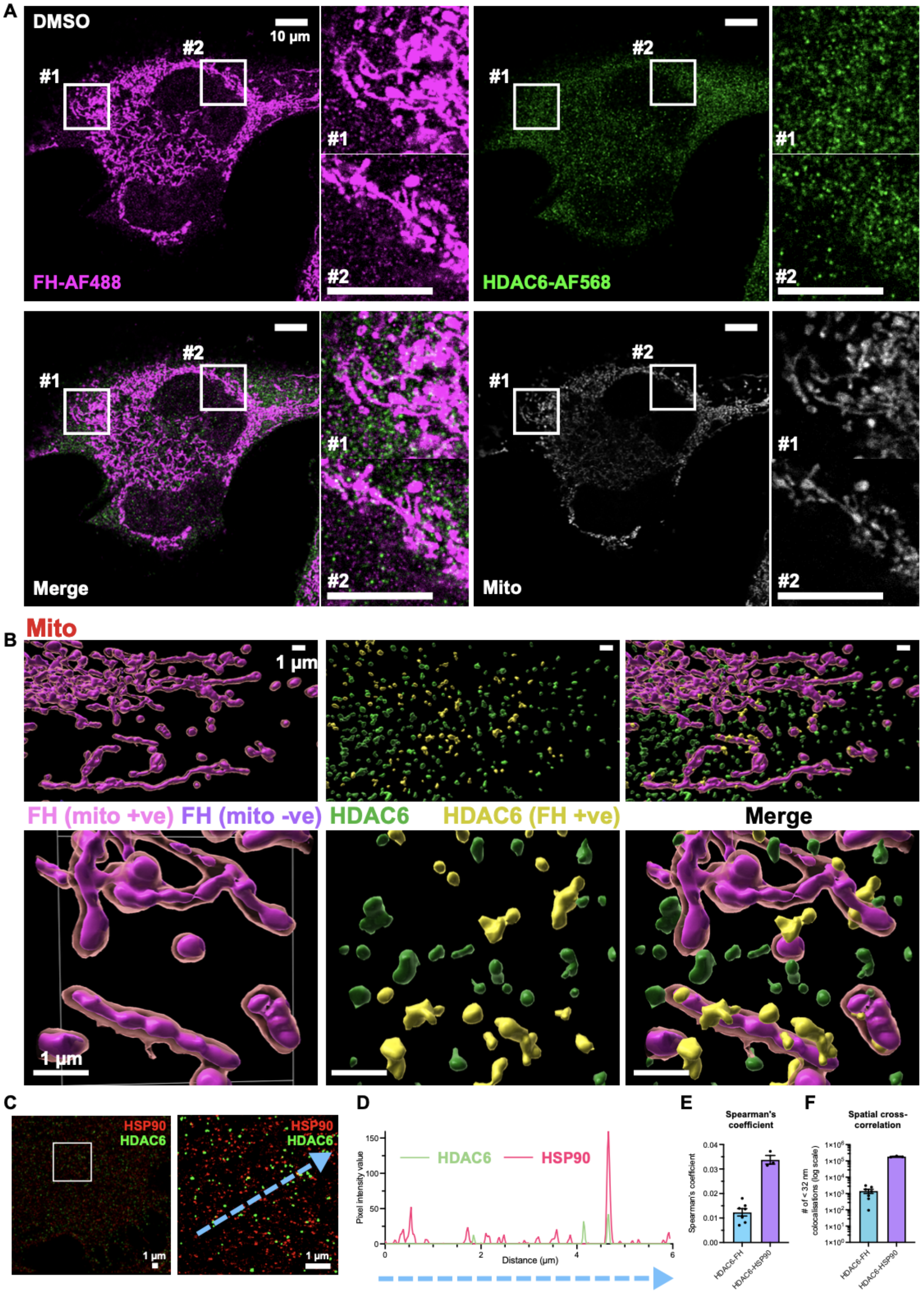


**Supplementary Figure 6. FH mostly localises to inside mitochondria indicated by MitoTracker staining and 3D immunofluorescence imaging in a BT-549 cell.**

(A) A representative single-plane image of a fixed BT-549 cell stained with MitoTracker DeepRed FM (grey) and immunostained for HDAC6 with AlexaFluor-568 (AF568, green) and FH with AlexaFluor-488 (AF488, blue). Scale bars, including inlay region, show 10 µm. (B) The region in (A) was used to reconstruct the cell in 3D. Mitochondrial volume is indicated in red. FH volume overlapping mitochondrial volume (FH mito +ve) is in magenta and FH not overlapping in dark purple. HDAC6 overlapping FH (HDAC6 FH +ve) is in yellow and not overlapping in green (HDAC6 FH -ve). A zoomed region is shown in the bottom panels. Scale bars show 1 µm. (C) Representative STORM visualisation of HDAC6 (green, AlexaFluor-647) and HSP90 (red, CF-680) expression in a BT-549 cell. (D) Line profile of the region indicated by the blue dotted line showing relative pixel intensity of HDAC6 and HSP90. (E-F) Colocalisation of HDAC6 and HSP90 (purple) as assessed by Spearman's correlation coefficient using Coloc2 (FIJI/ImageJ) or (F) spatial cross-correlation and compared to the colocalisation of HDAC6 and FH (blue). Each point represents z-stack regions of 20-30 images analysed together.

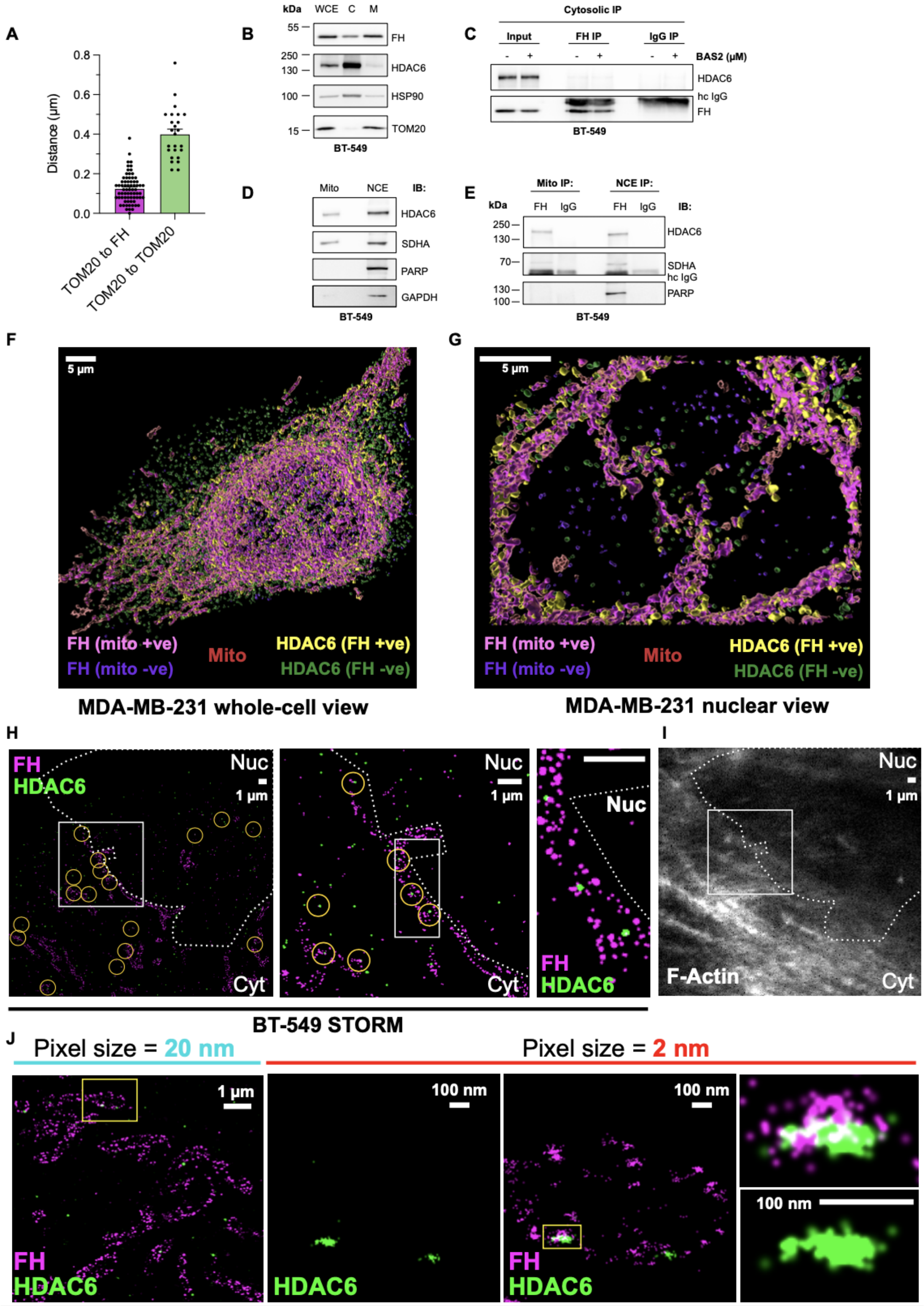


**Supplementary Figure 7. HDAC6 interacts with FH predominantly at mitochondria, as compared to the nucleus.** (A) Aggregated line profiles from STORM images. Mitochondrial

diameter is indicated by TOM20-to-TOM20 distance and FH distance to the mitochondrial membrane indicated by FH-to-TOM20 distance. (B) Representative Western blot of BT-549 cells showing cytosolic (C), mitochondrial (M), and whole-cell extract (WCE) fractions. (C) Representative Western blot showing HDAC6 expression in a purified mitochondrial fraction and in a nuclear cell extract (NCE). (D) Immunoprecipitation of FH in mitochondrial and NCE fractions from (C). (E) IP of FH in a nuclear extract in MDA-MB-231 cells and compared to the cytosol (cyt) fraction. (F) IP of FH in BT-549 cytosolic extractions after treatment with BAS-2 (10 μM) for 24 h. Non-specific bands from heavy-chain IgG (hc IgG) are indicated. (G) 3D reconstruction of mitochondria (MitoTracker DeepRed), HDAC6 (AlexaFluor-568), and FH (AlexaFluor-488) in a fixed MDA-MB-231 cell. Mitochondrial volume is indicated in red (Mito). FH volume overlapping mitochondria in magenta (FH mito +ve) and FH not overlapping mitochondria in dark purple (FH mito -ve). HDAC6 overlapping FH is in yellow (HDAC6 FH +ve) and not overlapping in green (HDAC6 FH -ve). (H) Representative STORM visualisation of a cytosolic and nuclear region of a BT-549 cell showing HDAC6 (AlexaFluor-647, green) and FH (CF-680, magenta). The nucleus (Nuc) was confirmed by negative F-actin staining (I) and is demarcated by the dotted white line. Yellow circles indicate overlapping pixels of HDAC6 and FH STORM localisations. Zoomed regions indicated by white boxes are sequentially shown. Scale bars show 1 μm. (J) STORM visualisation of HDAC6 (green) and FH (magenta) in a BT-549 cell first using a 20 nm pixel size on the left (blue). The region indicated by a yellow box is then shown using a smaller 2 nm (red) pixel size and the yellow-box region zoomed to the right. Scale bar shows 1 μm and 100 nm (zoomed regions).

**Figure S8**

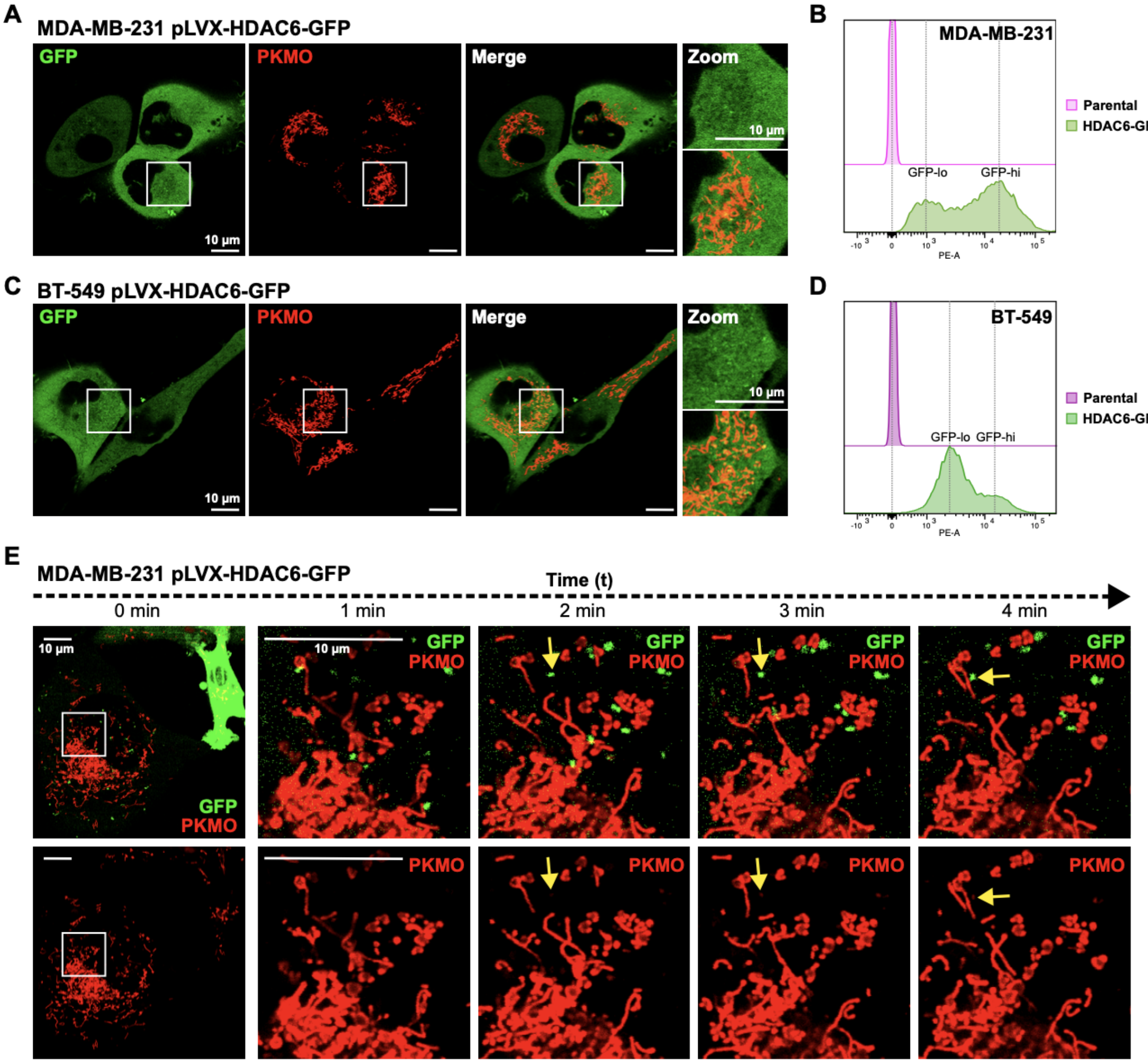


**Supplementary Figure 8. HDAC6-GFP associates with mitochondria.** (A) Representative live-cell confocal images of MDA-MB-231 cells stably expressing a lentiviral-transfected (pLVX) HDAC6-GFP vector (green) with cell-to-cell variability in the amount of GFP expression. Cells were stained with PKMitoOrange (PKMO, 300 nm, 30 min) to indicate mitochondria (red). Zoomed regions indicated by the white boxes are shown on the right. Scale bars show 10 µm (B) Representative flow cytometry histograms of parental or HDAC6-GFP-expressing MDA-MB-231 cells illustrating variable GFP expression. (C-D) As in (A-B) for the BT-549 cell line. (E) Confocal images from a 15 min time-lapse of live MDA-MB-231 cells showing HDAC6-GFP puncta after treatment with 10 µM BAS-2 for 24 h. Each frame indicates an interval of 1 min. Mitochondria were stained with PKMO and imaged using confocal microscopy. Arrows

indicate HDAC6 puncta associating with mitochondria. PKMO signal can be seen associating with the foci, indicating mitochondrial membranes. Scale bars show 5 µm.

**Figure S9**

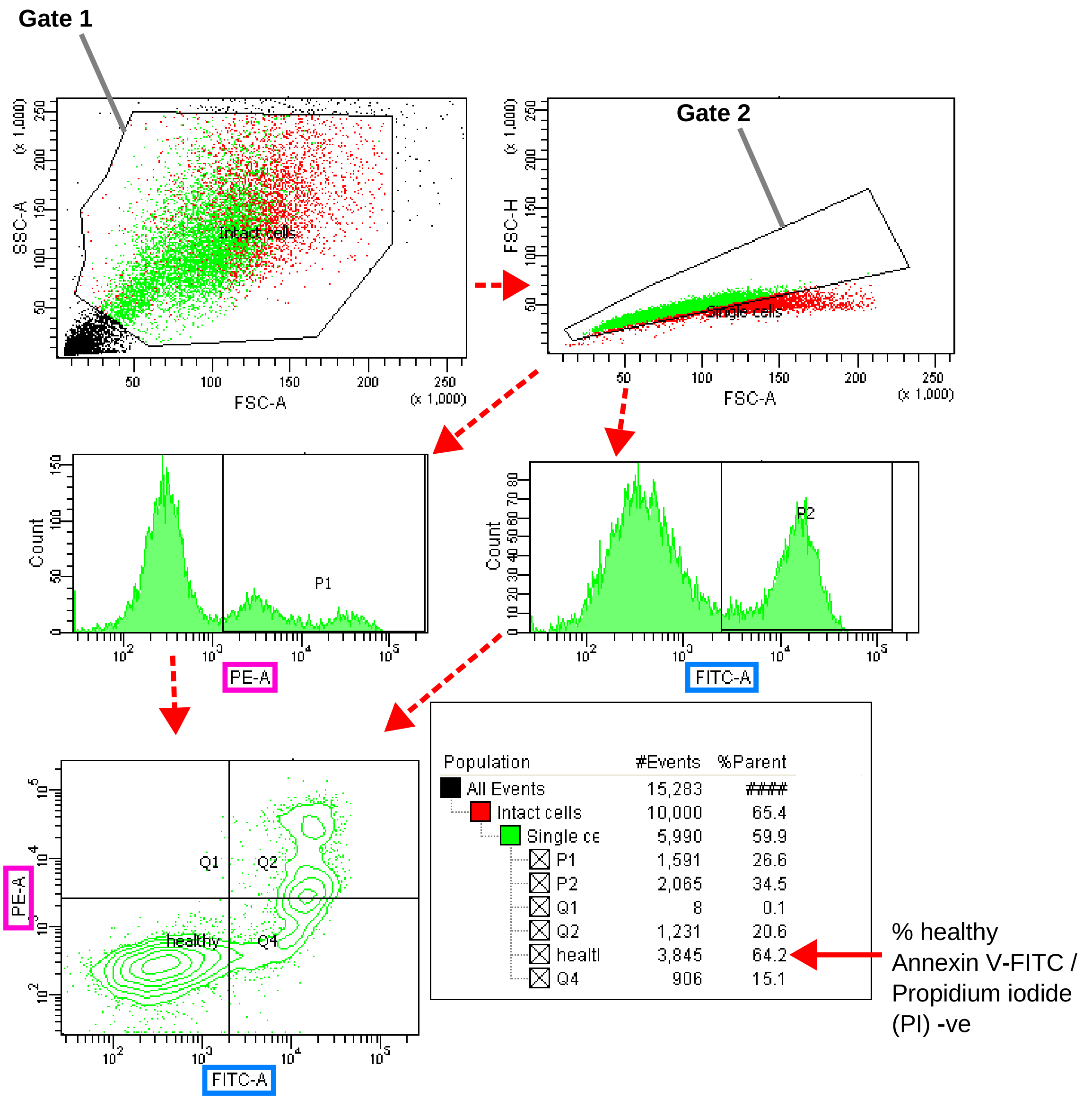


**Supplementary Figure 9. Gating strategy for Annexin V-FITC / propidium iodide (PI) flow cytometry to measure cell death.** Representative gating strategy of BT-549 cells after drug treatment. Cells were first gated (Gate 1) by forward scatter area (FSC-A) versus side scatter area (SSC-A) and secondarily gated (Gate 2) by FSC-A by forward scatter height (FSC-H). These gated events were then gated by quadrant using FITC (for Annexin V-FITC) and PE (for propidium iodide [PI]) settings. Events from Gate 2 that were negative for both Annexin V and PI were designated healthy and taken as a percentage of their parent. All other flow cytometry used Gates 1 and 2.

**Supplementary Figure 10. Uncropped blot images used for Supplementary Figures.**

**Fig S1B**
**MDA-MB-231**
**Ac-alpha-tubulin (K40)** **B-actin**

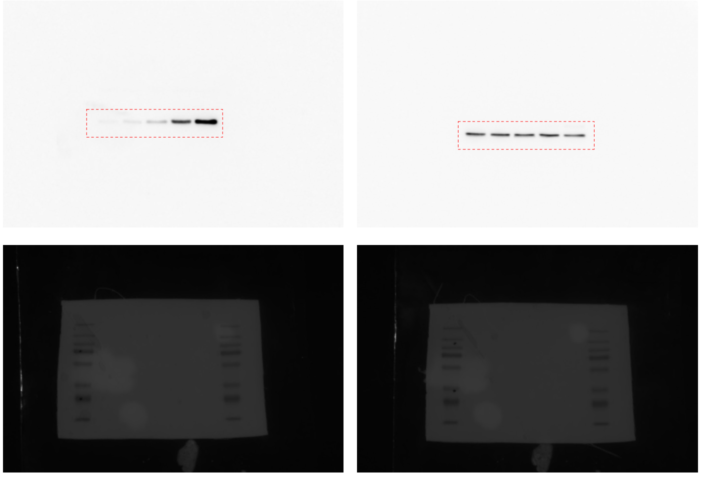

**Fig S1E**
**BT-549**
**Ac-alpha-tubulin (K40)** **B-actin**

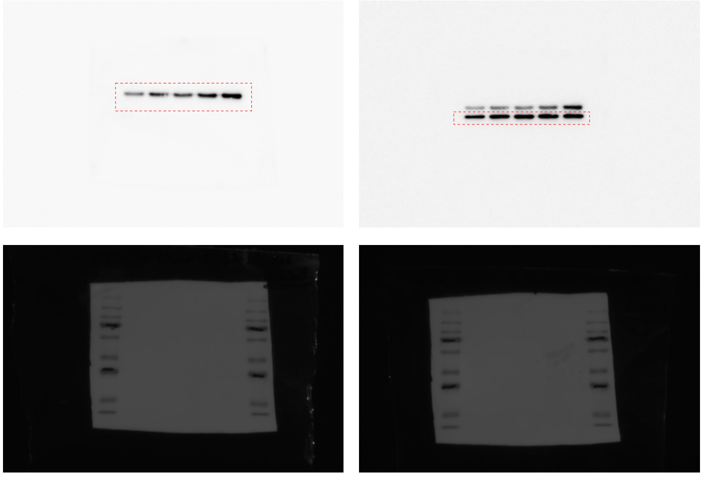

**Fig S2A & B**
**HDAC6** **HDAC6 (max adjusted)** **FH**

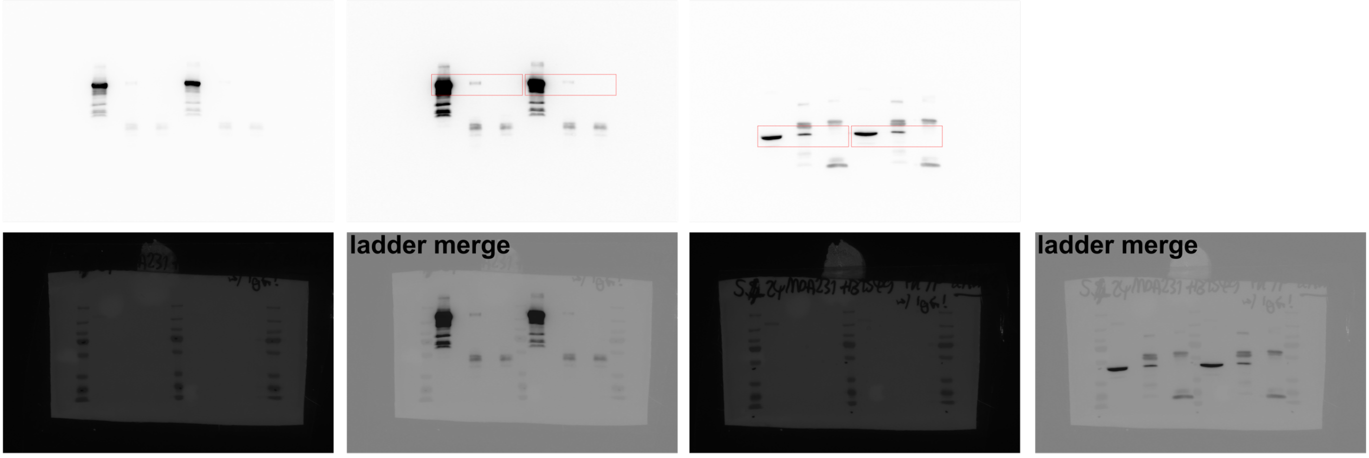

**Fig S2C** *HDAC6 IP* (run-through and antibody-only controls on right)

**FH** **FH (max adjusted)** **HDAC6**

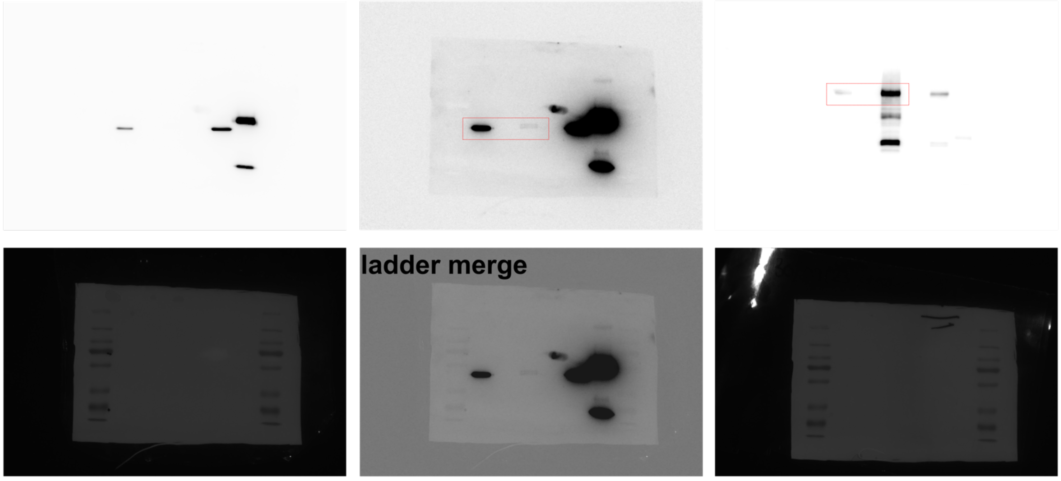


**Fig S2D**

**FH** **HDAC6**

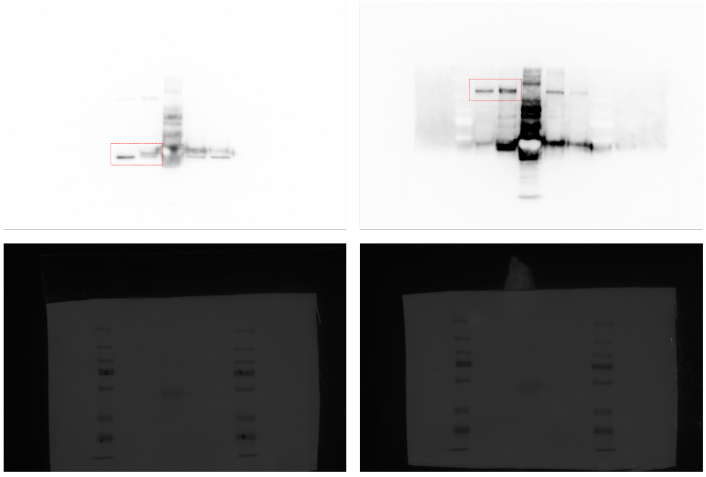

**Fig S2F** *FH IP*

**HDAC6**

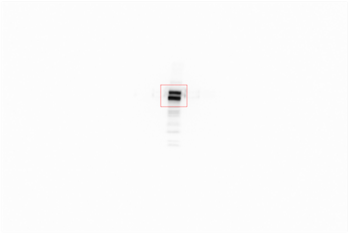

**HDAC6 (max adjusted)**

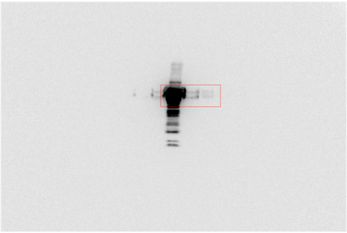

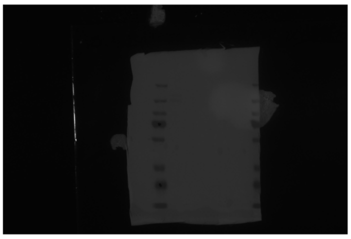

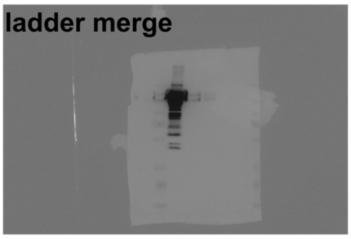


**Fig S2F** *FLAG IP*

**FLAG**



**FLAG (max adjusted)**

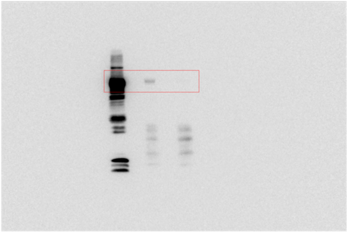

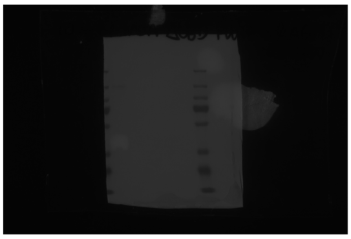

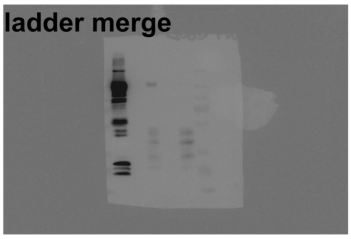


**Fig S2F**

**FH**

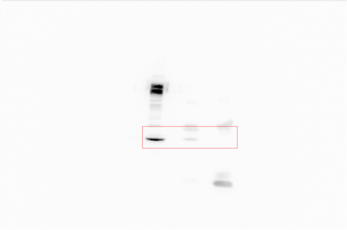

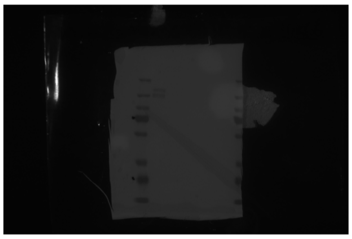

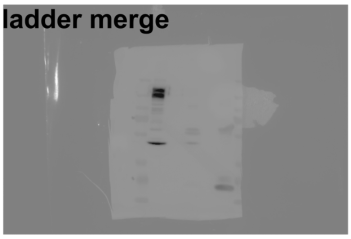


**FH**

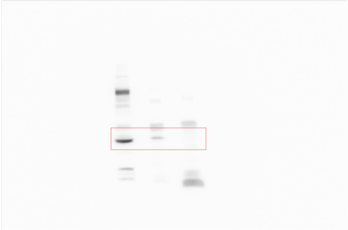

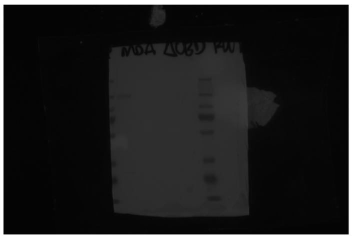

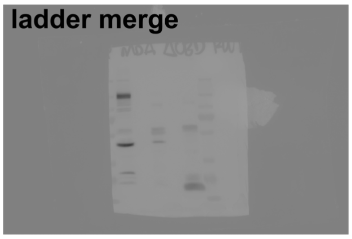

Fig S3F

MDA-MB-231

FH

Ac-alpha-tubulin (K40)

B-actin

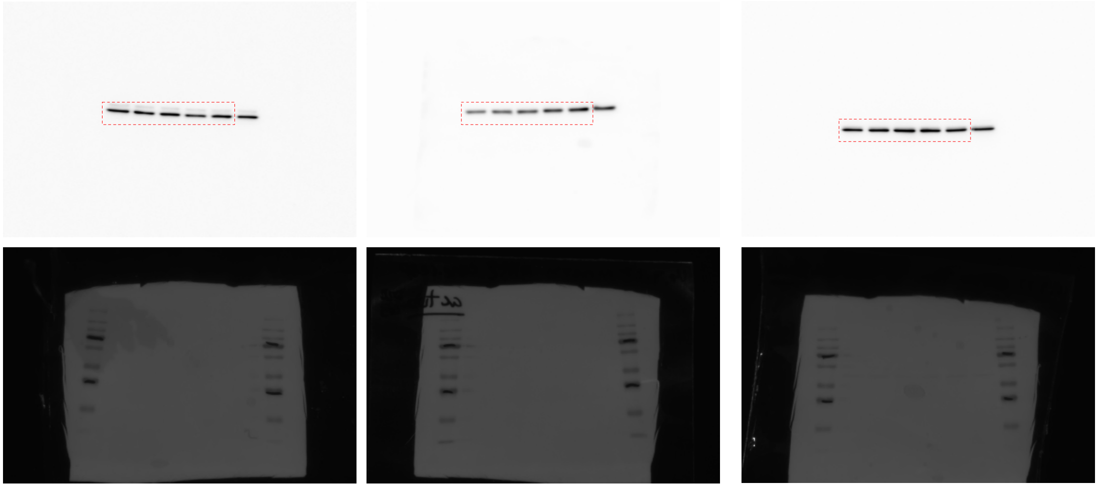

BT-549

FH

Ac-alpha-tubulin (K40)

B-actin

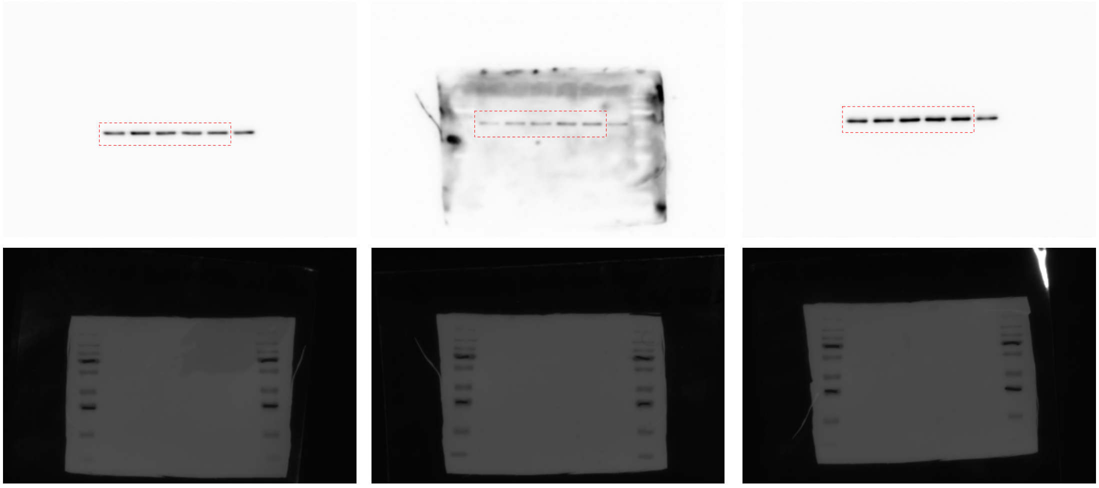

Fig S7

HDAC6

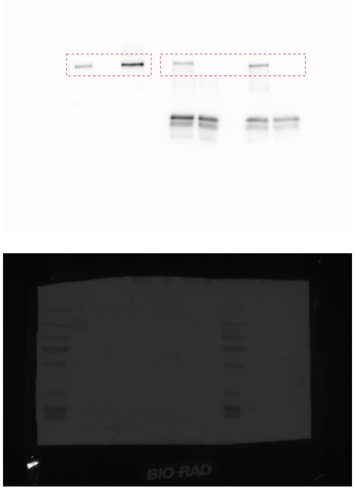

PARP

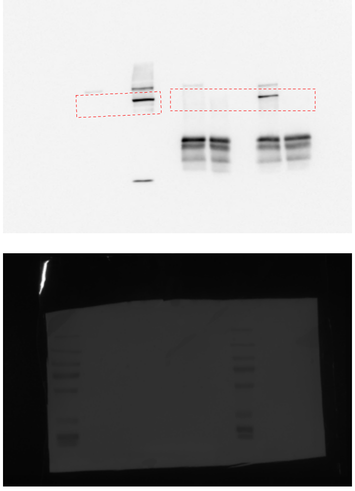

SDHA

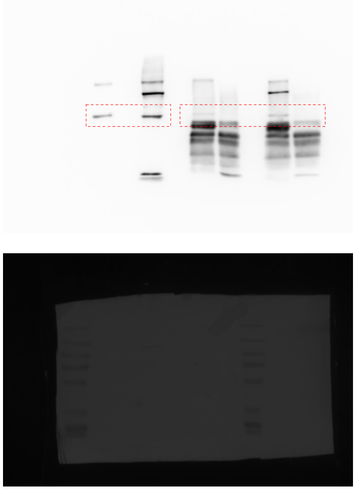

GAPDH

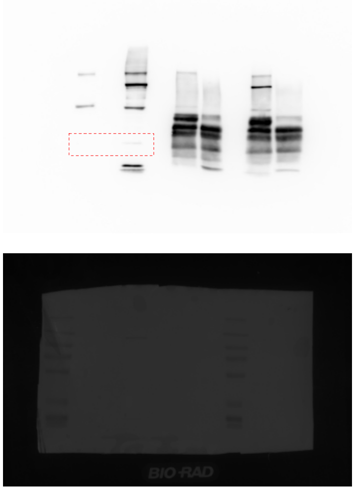

Fig S7

HDAC6

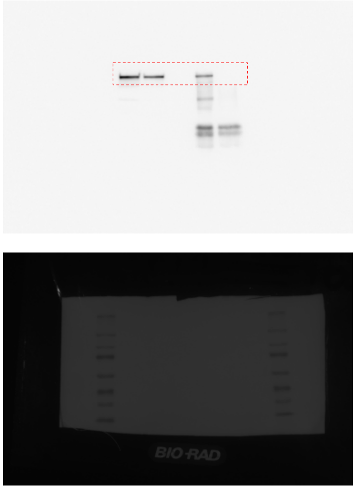

Histone 3

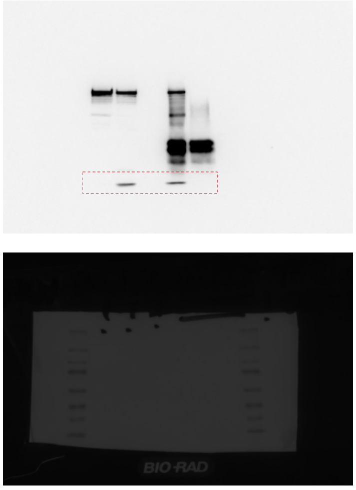

GAPDH

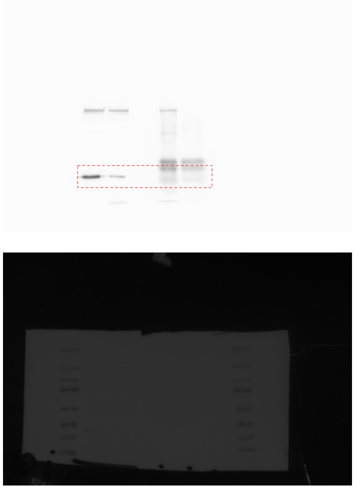

Fig S7

FH

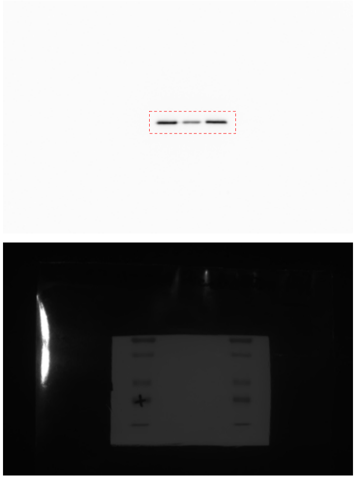

HDAC6

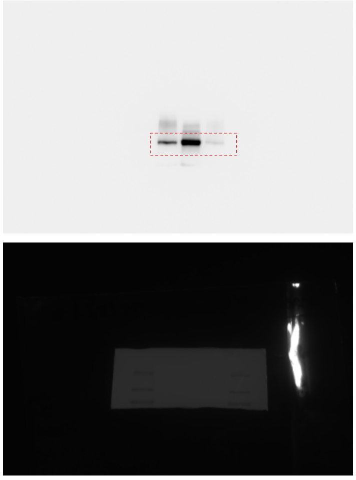

HSP90

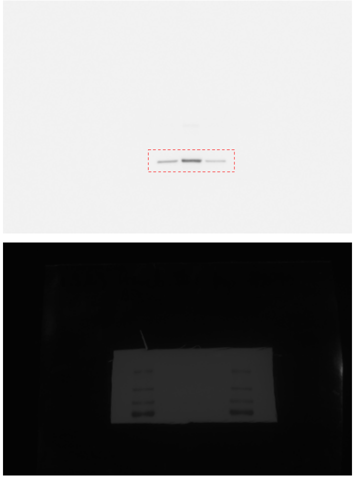

TOM20

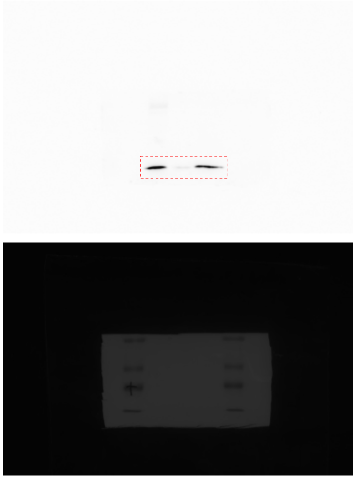

## Fig S7

HDAC6

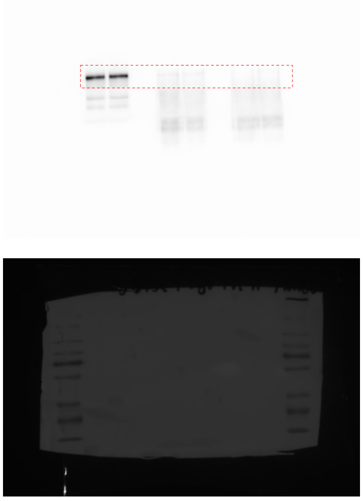

FH

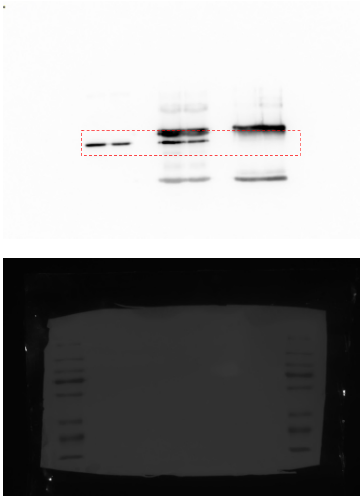

Supplement: Supplementary file 1 — Supplementary Information [file 41467_2025_61897_MOESM1_ESM.pdf]
