# Supplementary material for: Inhibition of HDAC6 alters fumarate hydratase activity and mitochondrial structure
Source: Nat Commun. 2025 Jul 28;16:6923. doi: 10.1038/s41467-025-61897-6 (PMC12304134; doi:10.1038/s41467-025-61897-6)
Supplement: Supplementary file 2 — Description of Additional Supplementary Files [file 41467_2025_61897_MOESM2_ESM.pdf]

Description of Additional Supplementary Files

File Name: Supplementary Movie 1
Description: Rotation of a fixed BT-549 cell shown in Supplementary Fig. 6 stained with MitoTracker DeepRed FM (red), HDAC6 (green), and FH (blue) reconstructed in 3D using a z-stack series.

File Name: Supplementary Movie 2
Description: 3Dvisualisation of a BT-549 cell region stained for mitochondria (red), FH (magenta/purple) and HDAC6 (yellow overlapping FH, green not overlapping). Surfaces rendered using Imaris and z-stack series of confocal images.

File Name: Supplementary Movie 3
Description: Video of a live MDA-MB-231 cell expressing HDAC6-GFP (green) and stained with PKMitoOrange (PKMO, red) for mitochondria. Cells were treated with BAS-2 for 24 h (10 µM). Stills are shown in Supplementary Fig 8E where 15 frames were taken at approximately 1-min intervals.
